# Molecular Insights into the Antistress Potentials of Brazilian Green Propolis Extract and Its Constituent Artepillin C

**DOI:** 10.3390/molecules27010080

**Published:** 2021-12-23

**Authors:** Ashish Kaul, Raviprasad Kuthethur, Yoshiyuki Ishida, Keiji Terao, Renu Wadhwa, Sunil C. Kaul

**Affiliations:** 1 AIST-INDIA DAILAB, DBT-AIST International Center for Translational & Environmental Research (DAICENTER), National Institute of Advanced Industrial Science & Technology (AIST), Tsukuba 305-8565, Japan; ashish-kaul@aist.go.jp (A.K.); raviprasadkv94@gmail.com (R.K.); 2 School of Integrative & Global Majors, University of Tsukuba, Tsukuba 305-8577, Japan; 3 Department of Cell and Molecular Biology, Manipal School of Life Sciences, Manipal Academy of Higher Education (MAHE), Manipal 576-104, India; 4 CycloChem Co., Ltd., 7-4-5 Minatojima-minamimachi, Chuo-ku, Kobe 650-0047, Japan; yoshiyuki.ishida@cyclochem.com (Y.I.); keiji.terao@cyclochem.com (K.T.)

**Keywords:** Brazilian green propolis, supercritical extract, stress inhibition, pro-hypoxia, neurodifferentiation

## Abstract

Propolis, also known as bee-glue, is a resinous substance produced by honeybees from materials collected from plants they visit. It contains mixtures of wax and bee enzymes and is used by bees as a building material in their hives and by humans for different purposes in traditional healthcare practices. Although the composition of propolis has been shown to depend on its geographic location, climatic zone, and local flora; two largely studied types of propolis: (i) New Zealand and (ii) Brazilian green propolis have been shown to possess Caffeic Acid Phenethyl Ester (CAPE) and Artepillin C (ARC) as the main bioactive constituents, respectively. We have earlier reported that CAPE and ARC possess anticancer activities, mediated by abrogation of mortalin-p53 complex and reactivation of p53 tumor suppressor function. Like CAPE, Artepillin C (ARC) and the supercritical extract of green propolis (GPSE) showed potent anticancer activity. In this study, we recruited low doses of GPSE and ARC (that did not affect either cancer cell proliferation or migration) to investigate their antistress potential using in vitro cell based assays. We report that both GPSE and ARC have the capability to disaggregate metal- and heat-induced aggregated proteins. Metal-induced aggregation of GFP was reduced by fourfold in GPSE- as well as ARC-treated cells. Similarly, whereas heat-induced misfolding of luciferase protein showed 80% loss of activity, the cells treated with either GPSE or ARC showed 60–80% recovery. Furthermore, we demonstrate their pro-hypoxia (marked by the upregulation of HIF-1α) and neuro-differentiation (marked by differentiation morphology and upregulation of expression of GFAP, β-tubulin III, and MAP2). Both GPSE and ARC also offered significant protection against oxidative stress and, hence, may be useful in the treatment of old age-related brain pathologies.

## 1. Introduction

Propolis is a complex mixture of resinous material, produced by bees by mixing their saliva with botanical sources they live on. It is an important structural component of beehives and chemical weapon of bees. Its color varies from yellowish green to dark brown, and odor from odorless to aromatic, and depends on its botanical source, origin of place, and bee characteristics such as strain and age [1]. Besides the structural and functional attributes of propolis for bees [2], it has been reported to possess a variety of therapeutic potentials for human use [3,4,5,6,7,8,9] since ancient times. There are mainly two kinds of propolis known that differ in their constituents: New Zealand propolis possesses CAPE (Caffeic Acid Phenethyl Ester) and Brazilian green propolis that possesses Artepillin C (3,5-diprenyl-4-hydroxycinnamic acid, ARC) as pre-dominant bioactive compounds [10,11,12]. Besides these major bioactives, a large variety of constituents including flavonoids, phenolic acids, esters, terpenoids, steroids, amino acids, and cinnamic acid derivatives have been identified in propolis and are considered as popular pharmacological research material [10,13]. Many studies have validated a broad spectrum of biological activities in propolis. These include anti-bacterial [14], anti-viral [15], anti-fungal [16], anti-inflammatory [17,18], and anti-tumor [19,20,21,22,23,24]. Most recently, propolis has also been used in cosmetic products and functional food/supplements. Molecular studies on the anticancer activity have revealed that the phenolic acid components of propolis including CAPE and ARC possess multi-modal anticancer activities that works through pathways including, mitochondrial stress [25], activation of tumor suppressor proteins [23,24,26], anti-inflammation activity [17,18,27,28], and activation of DNA damage signaling [23,24,29]. CAPE and ARC have been reported to differ in their bioavailability profile. Whereas CAPE has been shown to become degraded by secreted esterases [30], its complex with γCD was protected and showed improved activity in in vitro and in vivo anti-tumor assays [23]. ARC, on the other hand, has been shown to suffer from extremely low absorption efficiency and bioavailability [31]. Based on these aspects, propolis extracts with specific ingredients have become popular [32,33].

We had earlier performed cDNA array of CAPE-treated human cancer cells and found that the cytotoxicity of CAPE was mediated by activation of p53-GADD45 signaling. Bioinformatics and experimental evidence revealed that CAPE targets mortalin-p53 interactions, resulting in nuclear translocation and reactivation of p53 function leading to growth arrest in cancer cells [23]. Like CAPE, ARC also activated tumor suppressor activity in p53 by abrogating its complex with mortalin [33]. Several studies have demonstrated the supercritical extraction that uses high pressure, low temperature, and allows reduction in organic solvents as a preferred method of extraction for retaining sensitive natural bioactive compounds [34,35]. Supercritical extracts have been shown to retain aroma as well as bioactive profile that closely resembles the original source [36]. We had earlier prepared the Green Propolis Supercritical Extract (GPSE) and its complex with γCD (GPSE-γCD). By HPLC analysis, 0.5% GPSE was seen to contain ~9.6% ARC (equivalent to 16.6 μM). Cell culture assays, used to compare the cytotoxicity of GPSE with respect to pure ARC, showed anti-proliferation and anti-migration activities in 0.5% GPSE equivalent to ~500 μM of pure ARC [33]. In the present study, we investigated a wide range of dose-response and found that the low concentrations of GPSE (that lacked anti-proliferation and anti-migration activities) possess antistress potential. We experimentally prove this hypothesis using a variety of cell-based stress readouts including protein aggregation and misfolding, oxidative stress, and hypoxia. Furthermore, we discovered induction of pro-hypoxia and neuro-differentiation activities in response to the treatment. Taken together, these data predicted the potentials of ARC and GPSE in treatment and management of stress and old age-related pathologies.

## 2. Materials and Methods

### 2.1. Preparation of Green Propolis Supercritical Extract (GPSE)

Supercritical CO_2_ extraction offers an economic and non-cytotoxic way of extracting most natural compounds and, hence, is preferred over the organic solvent extractions. Green Propolis Supercritical Extract (GPSE) was prepared by conventional kneading method as reported earlier [33]. The extract was dissolved in dimethyl sulfoxide (DMSO) and kept on a shaker at room temperature for 2 h until it formed dark yellowish colored homogeneous liquid. The mixture was centrifuged at 160,000× *g* at 18 °C for 15 min after which the liquid supernatant was collected and again centrifuged. The clear supernatant obtained was carefully transferred to a fresh Eppendorf tube and used for further experiments. High-performance liquid chromatography (HPLC) using the Shimadzu HPLC system (LC-2010C; Shimadzu Corp., Kyoto, Japan) revealed the presence of ARC (~10%) along with other components of bee wax [33].

### 2.2. Cell Lines and Transfection Reagents

A549 (lung carcinoma), SKOV3 (ovarian carcinoma), and U2OS (osteosarcoma) cell lines were purchased from the Japanese Collection of Research Bioresources Cell Bank (JCRB) (Osaka, Japan). C6 (rat glioma) was obtained from the Cell Resource Center for Biomedical Research, Tohoku University, Sendai, Japan. All cells were cultured in DMEM (Dulbecco’s Modified Eagle’s medium) supplemented with 5% fetal bovine serum at 37 °C with 95% O_2_ and 5% CO_2_ in a humidified incubator. Lipofectamine 2000 (Invitrogen) in Opti-MEM (Gibco, Thermo Fisher Scientific, Waltham, MA, USA) media was used for transfection. Hypoxia responsive cells were obtained by stable transfection of plasmid encoding luciferase reporter driven by HIF-1α promoter as described earlier [37]. For heat shock induced misfolding of protein, cells transfected with plasmid (pGL4) encoding luciferase driven by a constitutive promoter were used [38]. For protein aggregation assays, GFP protein was used as a model. Cells expressing mortalin-GFP protein were generated by stable transfections of pEGFP-C1/mot-GFP plasmid. Sodium(meta)arsenite (NaAsO_2_), used in an induction of protein aggregation, was purchased from Sigma-Aldrich (St. Louis, MO, USA).

### 2.3. Cell Proliferation (MTT and WST) Assays

U2OS, A549, and SKOV-3 cells (5000/well) were seeded in a 96-well plate. Next day, treatment with different concentrations of GPSE extract were given for 48 h. DMSO was used as a solvent control of which the volume was matched to the amount used for respective GSPE- and ARC-treated cells in all the experiments. Cytotoxicity assay was performed using 3-(4,5-dimethylthiazol-2-yl)-2, 5-diphenyltetrazolium bromide (MTT) assay (Life Technologies/Thermo Fisher Scientific, Waltham, MA, USA) as described earlier [23]. Briefly, viability of control and treated cells was evaluated based on their metabolic activity as determined by the conversion of MTT (yellow) by the mitochondrial NADH dehydrogenases of living cells into formazan (purple). The statistical significance of the results was determined from three independent experiments including triplicate sets in each experiment. For WST assay, cells were treated with different concentrations of GPSE extract for 48 h followed by addition of premix WST-1 (Takara Bio Inc., Shiga, Japan). Cell viability (based on their metabolic activity) was measured at 450 nm with a reference wavelength at 630 nm.

### 2.4. Wound-Scratch Assay

U2OS, A549, and SKOV-3 cells (50,000/well) were seeded in a 6-well plate. Once they reached about ~90% confluency and made a monolayer, wound scratches were made in the middle of the well using a 200 μL tip. Each well was rinsed with PBS thrice to remove dead and floating cells. Non-cytotoxic dose of GPSE extract was added in the culture medium. The time of scratch was taken as 0 h. The scratched areas were photographed and measured at 0, 24, and 48 h under a phase contrast microscope.

### 2.5. Protein Aggregation and Disaggregation Assays

GFP aggregation reporter—U2OS cells (50,000/well) stably transfected with GFP plasmid were seeded on coverslips placed in 12-well plate and then allowed to settle overnight. The cells were subjected to heavy metal stress by incubating with sodium arsenite (20 μM) for 24 h, followed by washing with PBS and recovery in either GPSE- or ARC-supplemented medium for 48 h. The cells were fixed in acetone:methanol (1:1) on ice for 5 min, permeabilized with Triton-X in PBS (PBST) for 10 min followed by blocking with 2% bovine serum albumin protein dissolved in PBST for 2 h followed by incubation with Hoechst stain for 5 min. Cells were washed with PBST and Milli Q for 10 min each and mounted with FA Mounting Fluid (Pullman, WA, USA) on cover slips. The slides were viewed under a Zeiss Axioplan 2 microscope and images were taken using AxioCam camera (Carl Zeiss, Oberkochen, Germany).

### 2.6. Heat Induced Misfolding of Luciferase Reporter

U2OS cells (50,000/well) were seeded on coverslips, placed in a 6-well plate, and allowed to settle overnight. The cells were transfected with pGL4-p53-3′UTR expressing luciferase from a constitutive promoter as described earlier [38]. After 48 h, cells were heat-stressed at 42°C and 5% CO_2_ for 2 h, followed by recovery at 37°C either in control or drug-supplemented medium for the next 48 h. The cells were then lysed using passive lysis buffer to check for luciferase activity using the luciferase assay system (Promega, Madison, WI, USA, E1501) following the manufacturer’s protocol.

### 2.7. ROS Assay

U2OS cells (10,000/well) were plated in 12-well plate and allowed to settle overnight. Next day, cells were stressed with H2O2 (0.5 or 1 mM) for 2 h to induce reactive oxygen species followed by recovery in drug-supplemented medium for 24 h. Cells were then stained for ROS detection using Image-IT LIVE green ROS detection kit (Thermo Fisher Scientific, Waltham, MA, USA) following the manufacturer’s instructions.

### 2.8. JC-1 Staining

U2OS cells (50,000/well) were plated in a 12-well plate and allowed to settle overnight. The following day, cells were exposed with H_2_O_2_ (0.5 or 1 mM) for 2 h for disruption of mitochondrial membrane potential (ΔΨm) followed by recovery in drug-supplemented medium for 24 h. Control and treated cells were then stained with JC-1 dye (Abcam, Cambridge, UK; ab141387; 10 μg/mL), widely used as an indicator of mitochondrial membrane potential. Cells were incubated in JC-1 dye at 37 °C in CO_2_ incubator for 30 min as described earlier [39]. Cells were washed with PBS and immediately observed under AxioCam camera (Carl Zeiss, Oberkochen, Germany).

### 2.9. Cell Differentiation

C6 cells (20,000/well) were plated in 6-well plate and allowed to settle overnight followed by treatment with various stressors and recovery either in control or extract-supplemented medium. Cells were observed, at 5- and 10-day intervals, under a phase contrast microscope at 20× magnification and further processed for molecular analyses.

### 2.10. Western Blotting 

Cells (200,000/well) were seeded in a 6-well plate and allowed to settle overnight. Control and treated cells were harvested and lysed in RIPA buffer (FUJIFILM Wako Pure Chemicals Corp., Osaka, Japan) containing complete protease inhibitor cocktail (Roche Applied Science, Mannheim, Germany). The protein samples (20 µg) were separated in SDS-polyacrylamide gels, and electro-blotted onto PVDF membranes (Millipore, Burlington, MA, USA) using a semidry transfer blotter (Biometra, Tokyo, Japan). Western blot was performed with antibodies against MMP2 (Santa Cruz Biotechnology Inc., Dallas, TX, USA (Santa Cruz), SC-10736), MMP3 (Santa Cruz, SC-80202), MMP9 (Santa Cruz, SC-6840), hnRNP-K (Cell Signaling Technology, Inc. Danvers, MA, USA; #R332), Vimentin (Santa Cruz, SC-6260), Luciferase (Santa Cruz, SC-57604), GFAP (Sigma-Aldrich, St. Louis, MO, USA; G9269), NeuF-H (Cell Signaling Technology, Inc. Danvers, MA, USA; #2836), NCAM (Santa Cruz, SC-10735), MAP2 (Cell Signaling Technology, Inc. Danvers, MA, USA; #4542S), β-tubulin III (Abcam, Cambridge, UK; ab 18207), and HIF-1α (Abcam, Cambridge, UK; ab 51608). The blots were blocked with 3% BSA/TBST for 2 h and incubated with primary antibodies at 4 °C overnight. Next day, the blots were washed thrice with TBST and incubated with horseradish peroxidase conjugated with goat anti-mouse or anti-rabbit antibodies (Santa Cruz) and detected using ECL substrate (Amersham Pharmacia Biotech/GE Healthcare, Piscataway, NJ, USA). The blots were further probed with β-actin as an internal loading control. Protein expression was quantified using ImageJ 1.46 software (NIH, Bethesda, MD, USA).

### 2.11. Immunostaining

Cells (50,000/well) were seeded on coverslips placed in 12-well dish. Once the cells attached to the cover slips, they were treated with specific drugs for 48 h. The cells were then fixed with acetone:methanol (1:1), permeabilized with 0.1% Triton X-100 for 10 min and blocked with 2% bovine serum albumin (BSA) for 2 h, followed by incubation with primary antibodies (overnight) against Nestin (Santa Cruz, SC-23927), GFAP (Sigma-Aldrich, St. Louis, MO, USA; G9269), GAP43 (Santa Cruz, SC-33705), Vimentin (Santa Cruz, SC-6260), NeuF-H (Cell Signaling Technology, Inc. Danvers, MA, USA, 2836), NCAM (Santa Cruz, SC-10735), and HIF-1α (Novus Biologicals, Littleton, CO, USA; NB 100-479), washed with PBS-PBST-PBS for 10 min each. The cells were further incubated with either Alexa-488 or Alexa-594 (A11034 or A11032, Molecular Probes, Eugene, OR, USA) secondary fluorescent antibodies and counterstained with Hoechst 33258 (Sigma-Aldrich, St. Louis, MO, USA) before mounting. The slides were viewed under a Zeiss Axioplan 2 microscope and images were taken using AxioCam camera (Carl Zeiss, Oberkochen, Germany).

### 2.12. Statistical Analysis

All experiments were repeated for more than thrice. Statistical data from three or more independent experiments were expressed as mean ± standard deviation. Unpaired Student’s t-test (GraphPad Prism, online calculator) was performed to determine the statistical significance between the control and experimental samples. Values of *p* > 0.05 (ns), *p* ≤ 0.05 (*), *p* ≤ 0.01 (**), *p* ≤ 0.001 (***), and *p* ≤ 0.0001 (****) were considered non-significant, statistically significant, very significant, highly significant, and extremely significant, respectively.

## 3. Results

### 3.1. Low Doses of GPSE Are Non-cytotoxic

**Figure 1:**
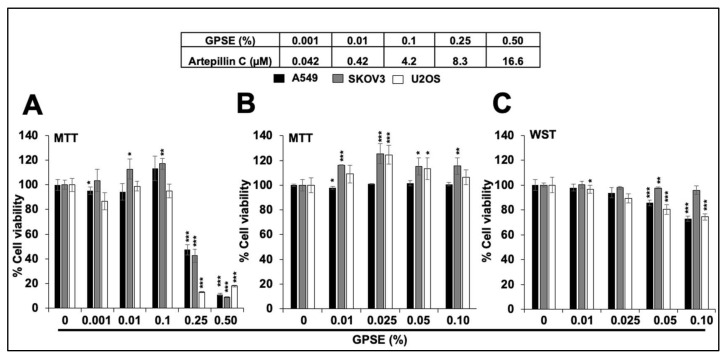
GPSE, at a low dose, is non-cytotoxic to human cancer cells. Dose dependent cytotoxicity of GPSE is shown. Effect of a wide range of GPSE concentrations (0.001 to 0.50%) showed cytotoxicity at the 0.25% and 0.50% in all the three cell lines (**A**). The low concentrations (0.001 to 0.10%) were confirmed to be non-cytotoxic by repeated MTT (**B**) as well as WST (**C**) assays. The quantified cell-viability data represents mean ± SD obtained from three independent biological replicates; *p*-values were calculated using unpaired Student’s *t*-test. * < 0.05, ** < 0.01, and *** < 0.001 represent significant, very significant, and very very significant, respectively.

**Figure 2:**
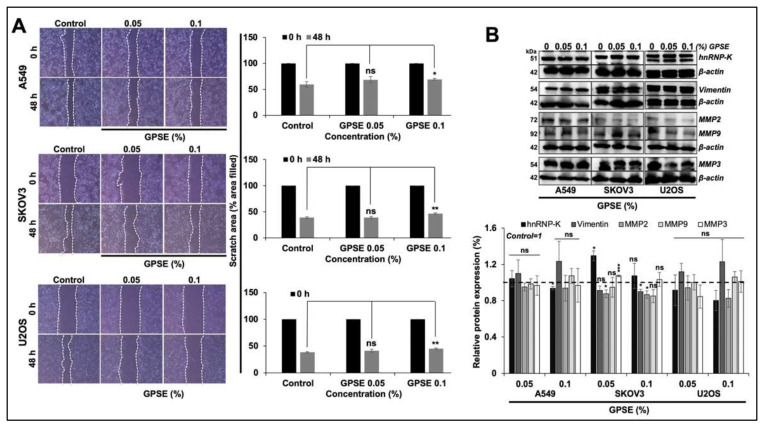
Low dose of GPSE did not affect the cell migration. Wound healing assay showing migration of cells in the wounded area. By 48 h and as examined in three cell lines there was no effect of GPSE (**A**). Western blotting of proteins (hnRNP-K, Vimentin, MMP2, MMP9, and MMP3) involved in cell migration did not show any change in the expression level in control and treated cells (**B**). The quantified data represents mean ± SD obtained from three independent biological replicates; *p*-values were calculated using unpaired Student’s *t*-test. * < 0.05, ** < 0.01, and *** < 0.001 represent significant, very significant, and very very significant, respectively. ns = not significant.

We investigated the concentration-dependent cytotoxicity of GPSE in three human cancer (A549, SKOV3, and U2OS) cell lines. As shown in Figure 1A, whereas 0.25 and 0.50% GPSE caused remarkable reduction in viability of all the three cell lines, low doses (0.001 to 0.10%) were non-cytotoxic. We confirmed these data by several independent experiments using MTT and WST assays (Figure 1B,C). We speculated that the low non-toxic doses of GPSE may possess anti-migration activity, useful for treating the metastatic phenotype of cancer cells. Standard wound-scratch assay was used to determine the effect of low non-cytotoxic concentrations (0.05 and 0.1%) of GPSE on the cell migration characteristics in three cells lines. As shown in Figure 2A, neither of these two doses were seen to alter migration characteristics in any of these cell lines. Furthermore, the key regulator of migration did not show any change on the Western blots (Figure 2B). These data confirmed that the low doses of GPSE neither affect proliferation nor migration characteristics of cells.

### 3.2. Non-Cytotoxic Doses of GPSE and Arc Inhibited Metal- and Heat-Induced Protein Aggregation 

**Figure 3:**
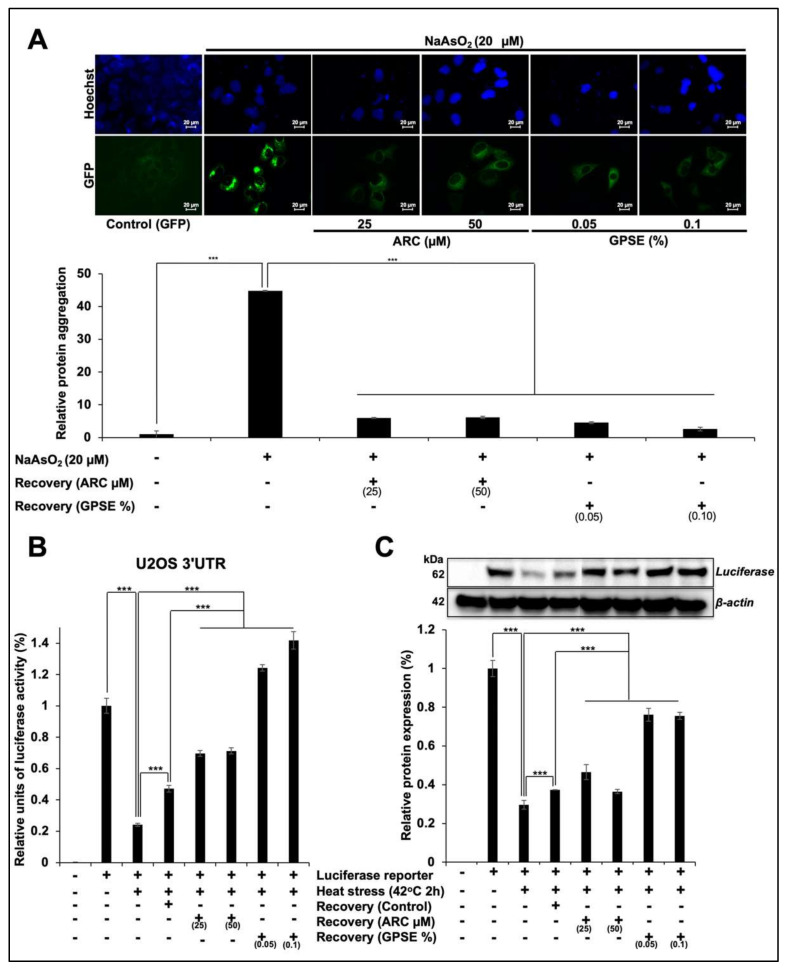
Low dose of GPSE protected human cells against metal stress. Cells stably transfected with GFP plasmids showed diffuse green fluorescence in the cytoplasm. Cells subjected to metal stress showed aggregation of GFP fluorescence, while the ones recovered in either GPSE- or ARC- supplemented medium showed recovery from aggregation (**A**). Heat-stressed cells showed 80% reduction in luciferase reporter activity that was recovered by 20–30% following replacement with fresh medium. Cells recovered in ARC- or GPSE-supplemented medium showed ~50 or 100% recovery in luciferase, respectively (**B**). Western blotting of cells transfected with luciferase plasmid showed 62-kDa luciferase protein band. The level of expression of luciferase reduced by heat stress and showed recovery when cells were cultured in ARC- or GPSE-supplemented medium (**C**). The quantified data represents mean ± SD obtained from three independent biological replicates; *p*-values were calculated using unpaired Student’s *t*-test. *** < 0.001 represent significant, very significant, and very very significant, respectively.

Anticipating that the low non-cytotoxic doses of GPSE and its active ingredient (ARC) may have antistress activity, we investigated their effect in metal-induced toxicity assays. We performed protein aggregation-disaggregation assay on control, metal-stressed, and ARC/GPSE-treated cells using GFP fluorescence as a reporter. U2OS cells stably transfected with GFP plasmid showed GFP fluorescence under the microscope. Of note, NaAsO2-treated cells showed aggregation of the GFP fluorescence (Figure 3A).

On the other hand, cells treated with ARC as well as GPSE showed diffused GFP fluorescence. Visual quantitation of cells revealed 40% cells with aggregated protein in response to NaAsO_2_ treatment. On the other hand, ARC- or GPSE-treated cells showed only 3–5% cells with aggregated protein suggesting that both these treatments could cause disaggregation of metal-induced aggregation of proteins (Figure 3A).

We also confirmed the above phenomenon by using heat induced misfolding/aggregation of luciferase protein. Cells transfected with luciferase reporter were exposed to heat stress followed by recovery either in control or ARC/GPSE-supplemented medium. As shown in Figure 3B, heat-stressed and recovered (control medium) cells showed ~80% and ~60% reduction in luciferase activity, respectively. On the other hand, cells recovered in ARC (25 μM)-supplemented medium showed 10–15% better recovery as compared to the cells recovered in control medium. Of note, cells recovered in GPSE-supplemented medium showed remarkably better (40% higher as compared to the control) recovery in luciferase activity. The results were confirmed by several independent experiments and by Western blotting of control, stressed and recovered cells (Figure 3C). Whereas a sharp reduction in luciferase protein was observed in heat-stressed cells, ARC- and GPSE-treated cells showed recovery in the luciferase protein. Taken together with the reporter assays, these data showed GPSE-treated cells possessed higher level of expression of luciferase protein as compared to the stressed-control as well as ARC treated cells.

### 3.3. Non-Cytotoxic Doses of GPSE and ARC Promoted Hypoxia and Differentiation Signaling 

**Figure 4:**
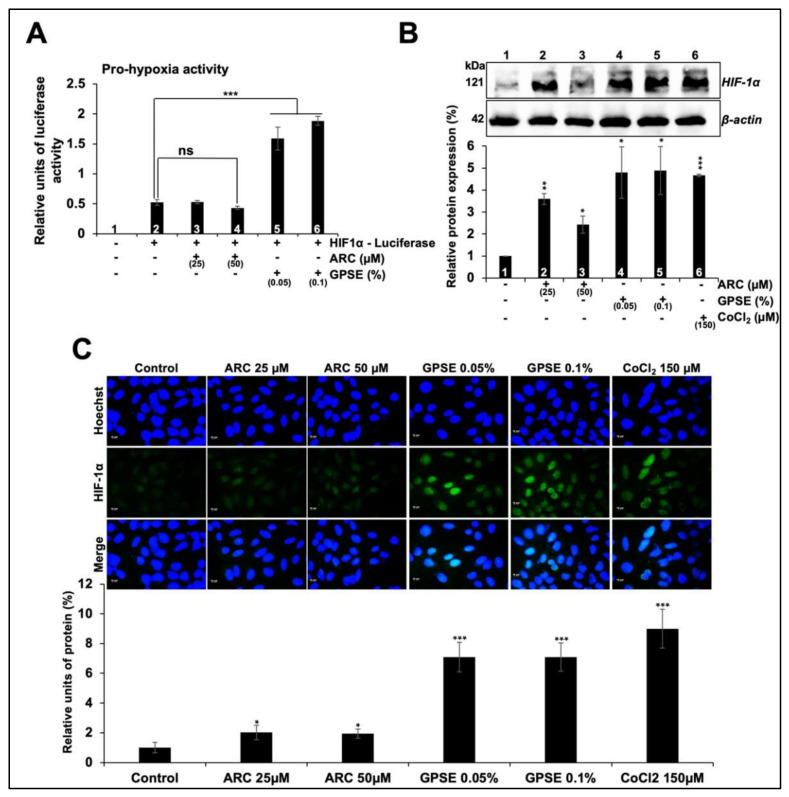
Low dose of GPSE caused activation of hypoxia signaling. Cells stably transfected with HIF-1α driven luciferase were treated with either ARC or GPSE. While ARC-treated cells did not show any change, GPSE-treated cells showed activation of hypoxia signaling (**A**). Western blotting of control and treated cells showed upregulation of endogenous HIF-1α in ARC and GPSE-treated cells (**B**). Immunostaining of control and treated cells showed upregulation of HIF-1α in ARC- and GPSE-treated cells. CoCl_2_ was used as a positive control for chemical induction of hypoxia (**C**). The quantified data represents mean ± SD obtained from three independent biological replicates; *p*-values were calculated using unpaired Student’s *t*-test. * < 0.05, ** < 0.01, and *** < 0.001 represent significant, very significant, and very very significant, respectively. ns = not significant.

Protein aggregation is commonly associated with hypoxia and age-related brain pathologies. In this context, we next examined if GPSE and ARC could upregulate hypoxia signaling at the molecular level. We used luciferase reporter assay driven by hypoxia inducible factor (HIF-1α). As shown in Figure 4A, U2OS cells treated with GPSE showed increase in HIF-1α driven luciferase activity. Furthermore, both Western blotting and immunostaining of control and treated cells with HIF-1α specific antibody showed upregulation of endogenous HIF1-α expression (Figure 4B,C). Of note, GPSE caused stronger upregulation as compared to ARC. Considering the above findings, we hypothesized that the induction of hypoxia signaling may be useful for treatment of pathologies in which hypoxia and protein aggregation play a major role.

**Figure 5:**
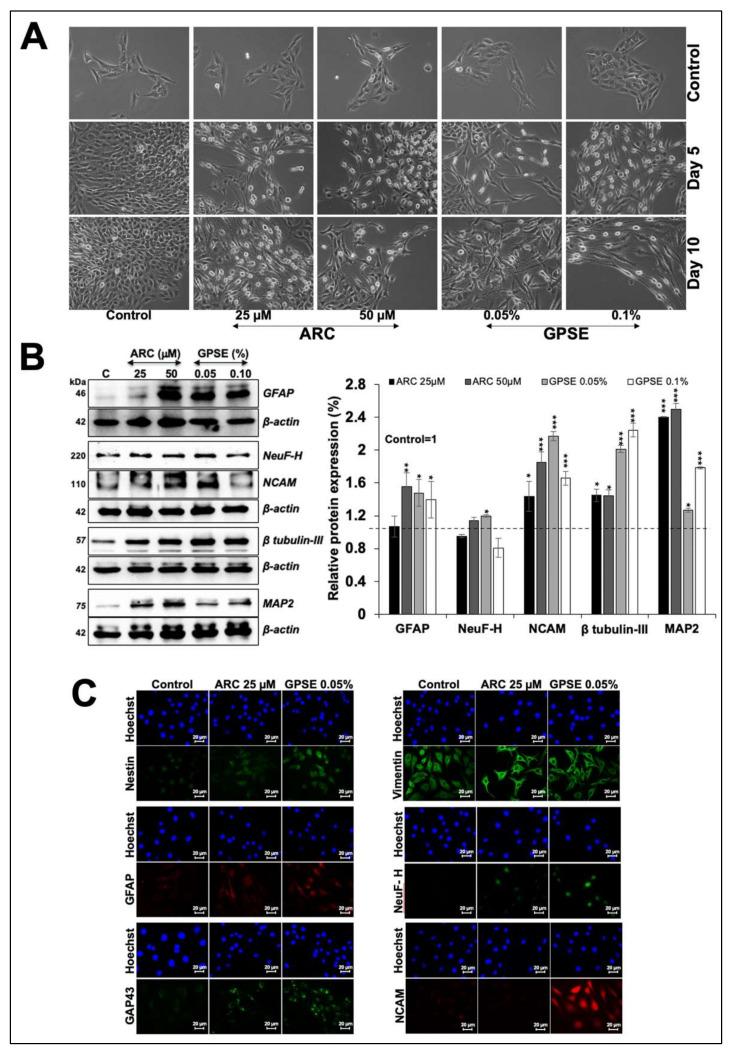
Low dose of GPSE caused astrocytic differentiation: C6 cells treated with ARC or GPSE showed astrocytic morphology (**A**). Western blotting of control and treated cells showed increase in proteins involved in differentiation. Quantitation of the proteins from three independent experiments is shown on the right (**B**). Immunostaining showed upregulation of proteins involved in C6-differentiation (**C**). The quantified data represents mean ± SD obtained from three independent biological replicates; *p*-values were calculated using unpaired Student’s *t*-test. * < 0.05, ** < 0.01, and *** < 0.001 represent significant, very significant, and very very significant, respectively.

In this context, we next used brain-derived cells to examine the effect of GPSE and ARC on their differentiation properties. C6 glioma cells exposed to GPSE, and ARC for 5–10 days showed progressive increase in the number of differentiated cells with astrocytic morphology (Figure 5A). Western blotting and immunostaining showed increase in molecular markers (GFAP, NeuF-H, β-tubulin-III, MAP2, Nestin, and GAP43) that confirmed the differentiation of glioma to astrocytes (Figure 5B,C). Consistent with the stronger induction of differentiation morphology, most of the molecular markers showed higher level of expression in GPSE-treated cells as compared to the control and ARC-treated cells.

### 3.4. Non-Cytotoxic Doses of GPSE and ARC Have Potential to Protect against Oxidative Stress

**Figure 6:**
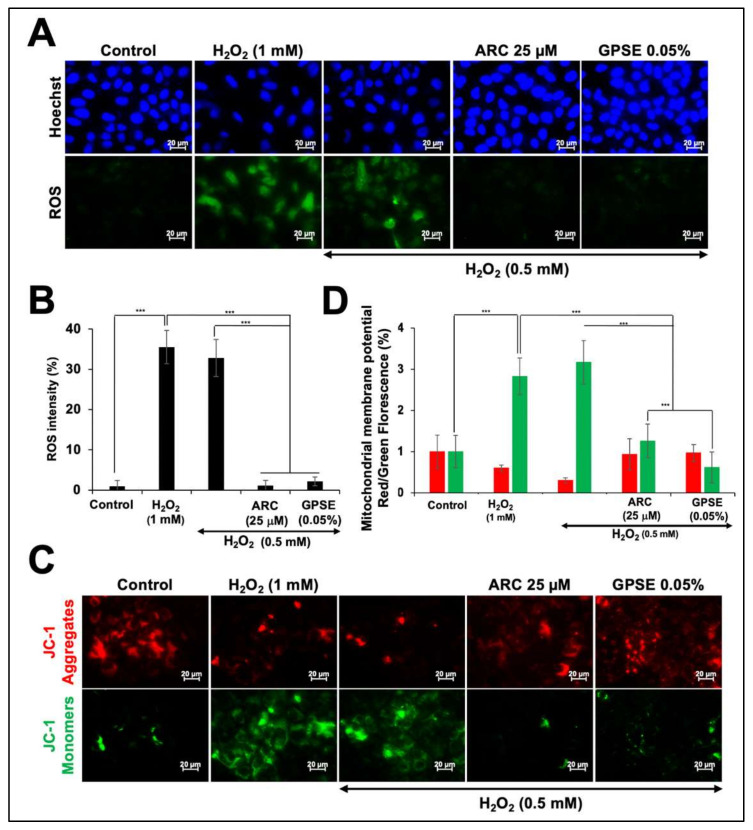
Low dose of GPSE protected the cells against oxidative stress: Control and treated cells subjected to ROS and JC-1 staining assays showed increased in ROS with H_2_O_2_-induced oxidative stress and its remarkable decrease in ARC- and GPSE-treated cells (**A**,**B**). JC-1 staining as examined by JC-1 dye (ab141387) showed loss of mitochondrial potential, as indicated by decrease in red staining, in cells subjected to oxidative stress by H_2_O_2_. ARC and GPSE caused protection against oxidative stress-induced loss of mitochondrial membrane potential (**C**,**D**). The quantified data represents mean ± SD obtained from three independent biological replicates; *p*-values were calculated using unpaired Student’s *t*-test. *** < 0.001 represent significant, very significant, and very very significant, respectively.

Since upregulation of reactive oxygen species (ROS) is tightly associated with oxidative stress and molecular damage that mark old age-related pathologies, we examined the level of ROS in control and treated cells. As shown in Figure 6A,B, cells exposed to H_2_O_2_ showed 15–20% increase in ROS. Of note, recovery of cells either in ARC- or GPSE-supplemented culture medium caused reduction in ROS level. We also performed JC-1 staining to determine the mitochondrial membrane potential in control and treated cells. As shown in Figure 6C,D, control cells showed red staining indicative of JC-1 polymers and high mitochondrial membrane potential. Oxidatively-stressed cells showed decrease in red and increase in green staining indicative of low mitochondrial membrane potential and JC-1 monomers. On the other hand, cells treated with either ARC or GPSE showed remarkable increase in JC-1-red staining. These data suggested that GPSE and ARC possess strong antioxidative potential and contribute to maintain mitochondria membrane potential, an essential component in the process of energy storage during oxidative phosphorylation and hence determines the mitochondrial and cell health. Based on these data that showed protective effect of ARC and GPSE on oxidative state of cells, they were predicted to be helpful in the management of a variety of old-age related pathologies, driven by oxidative stress.

## 4. Discussion

Oxidative stress is an inevitable outcome of oxidative metabolism that yields energy and is mandatory life-driving force. It is often quantitated as the level of ROS and offers a convenient assay to access antistress potential of natural and synthetic compounds. ROS homeostasis is achieved as a result of activities of several enzymes and natural antioxidants [40], and their imbalance has been connected to macromolecular damage leading to a variety of chronic illnesses including neurodegenerative disorders, metabolic disorder, and cancer [41,42,43]. Various kinds of traditional home medicine components have been shown to inhibit ROS production and impart protective role against neuronal injury [44,45]. In carbon tetrachloride (CCl4)-induced liver fibrosis mice model, mice treated with CCl4 showed collagen deposition in liver, and pathological alterations in spleen and lymph node. Furthermore, secondary lymphoid organs showed a significant increase in the circulation of T and B cells as well as intracellular levels of TGF-β, Nrf2, COX-2, eNOS, ROS, NO, and proinflammatory cytokines. Propolis treatment caused substantial suppression of liver collagen deposition, inflammatory signaling cascades suggesting its benefits against fibrotic complications and cancer [46]. Cancer chemotherapy has been shown to induce cognitive dysfunction by induction of oxidative stress and neuroinflammation. In the mice model of Chemo brain that shows cancer related cognitive impairment, effect of CAPE (pro-oxidant in cancer cells, but a potent antioxidant and cytoprotective in normal cells) was investigated. Learning and memory functions as determined by Morris water maze and passive avoidance tests showed that whereas chemotherapeutic drug (Doxorubicin) caused significant increase in oxidative stress, neuroinflammation, and impairment in learning and memory, co-treatment with CAPE counteracted Doxorubicin-induced such behavioral and molecular abnormalities in rat brain tissues [47]. Furthermore, CAPE was shown to suppress the growth of melanoma (most serious skin cancers that often show drug resistance and high metastatic ability) cells by induction of oxidative stress. Electroporation of melanoma cells with CAPE was suggested as an efficient delivery system [48]. In line with these reports, we found that like CAPE, whereas high doses of ARC offer anticancer activity [33], low doses possess antistress (oxidative, metal, and hypoxia) activity.

To enhance the efficacy of bioactive substances in propolis extracts, several studies have reported extraction protocols using a variety of solvents. Spanidi et al. [49] have reported a new controlled release system for propolis polyphenols. The antioxidant, antimutagenic and anti-aging properties of the system were investigated under normal and UVB-induced oxidative stress conditions in cultured skin cells and reconstituted skin model. It was shown that the system possesses high polyphenol encapsulation efficiency, physicochemical stability as well as controlled release rate in appropriate conditions. We had earlier reported that the CAPE-γCD complex possessed higher stability and anticancer activity in in vitro and in vivo assays [23,47,50]. Mortalin-targeting CAPE nanoparticles (CAPE-MotAb), generated to enhance the specific targeting of CAPE to cancer cells, caused stronger cytotoxicity and anti-migratory activity in cancer cells [19]. We earlier reported pro-hypoxia and antistress activities in the low doses of CAPE [37] that were also attributed to its potent neurodifferentiation activity in in vitro and in vivo mouse model [42]. Similarly, ARC has been reported to prevent dose-dependent oxidative damage, and cause inhibition of lipid peroxidation by 16% (as evaluated with thiobarbituric acid reactive substances) and by 36% (as evaluated with the formation of 8-hydroxy-2′-deoxyguanosine in DNA). Based on these data, ARC has been considered as a bioavailable antioxidant [51]. In another study, supercritical extract of Brazilian green propolis was shown to be more effective than the pure ingredient, ARC [33]. Oxidative stress and synapse dysfunction are the major neurodegenerative damage correlated to cognitive impairment in old age-related pathologies including Alzheimer’s disease (AD). Ni et al. [52] reported beneficial effects of Brazilian green propolis against neurodegenerative damage. These included improvement in cognitive functions of patients with mild cognitive impairment at high altitude. Using human neuroblastoma cells as a model, they attributed the effects to anti-oxidative properties of propolis, expression of brain-derived neurotrophic factor (BDNF), and activity-regulated cytoskeleton-associated protein (Arc), as being the critical factors of synapse efficacy. Similarly, Park et al. [53] reported an increase in viability of ischemic retinal ganglion cells (RGCs) treated with Brazilian green propolis. It was also shown to protect against RGC loss in ischemic retina. The mechanism of protection involved increase in HIF-1α, GFAP and histone acetylation. On the other hand, downregulation of apoptotic stimuli, and suppression of NF-kappaB-mediated inflammatory signaling was recorded. In continuation with these studies, we report here that the low concentrations of supercritical extract of green propolis (GPSE) and ARC possess beneficial antistress activities that may be useful for prevention and therapy of a variety of stress-related diseases, and hence warrant further experimental and clinical attention.

## 5. Conclusions

By a variety of cell-based assays, we demonstrate that the low non-cytotoxic doses of supercritical extract of Brazilian green propolis and Artepillin C possess antistress potentials. They offered protection against oxidative, protein aggregation, and hypoxia stresses. Furthermore, they promoted differentiation of brain-derived cells. The data suggested that the extract and the Artepillin C could be useful in treating protein aggregation- and hypoxia-related disorders, commonly associated with old age-related pathologies.

## Data Availability

The authors confirm that the data supporting the findings of this study are available within the article and/or its Supplementary Materials.

## Supplementary Materials

The following supporting information can be downloaded. Uncropped Western blots of Figure 2, Figure 3, Figure 4 and Figure 5. Figure S1: Western blot of protein of interest (hnRNP-K) in three cell lines A549, SKOV3, U2OS and its respective β-actin expression presented in Figure 2B; Figure S2: Western blot of protein of interest (Vimentin) in three cell lines A549, SKOV3, U2OS and its respective β-actin expression presented in Figure 2B; Figure S3: Western blot of protein of interest (MMP2) in three cell lines A549, SKOV3, U2OS and its respective β-actin expression presented in Figure 2B; Figure S4: Western blot of protein of interest (MMP3) in three cell lines A549, SKOV3, U2OS and its respective β-actin expression presented in Figure 2B; Figure S5: Western blot of protein of interest (MMP9) in three cell lines A549, SKOV3, U2OS and its respective β-actin expression presented in Figure 2B; Figure S6: Western blot of protein of interest (Luciferase) and its respective β-actin expression presented in Figure 3C; Figure S7: Western blot of protein of interest (HIF-1α) and its respective β-actin expression presented in Figure 4B; Figure S8: Western blot of proteins of interest (GFAP and NeuF-H) and their respective β-actin expression presented in Figure 5B; Figure S9: Western blot of proteins of interest (NCAM and β tubulin) and their respective β-actin expression presented in Figure 5B; Figure S10: Western blot of protein of interest (MAP2) and its respective β-actin expression presented in Figure 5B.

## References

[1] Kuropatnicki A.K., Szliszka E., Krol W. (2013). Historical Aspects of Propolis Research in Modern Times. Evid. Based Complement. Altern. Med..

[2] Bankova V. (2005). Chemical diversity of propolis and the problem of standardization. J. Ethnopharmacol..

[3] Ramos A.F.N., Miranda J.L. (2007). Propolis: A review of its anti-inflammatory and healing actions. J. Venom. Anim. Toxins Incl. Trop. Dis..

[4] da Silveira C.C.S.M., Luz D.A., da Silva C.C.S., Prediger R.D.S., Martins M.D., Martins M.A.T., Fontes-Júnior E.A., Maia C.S.F. (2021). Propolis: A useful agent on psychiatric and neurological disorders? A focus on CAPE and pinocembrin components. Med. Res. Rev..

[5] Kurek-Górecka A., Górecki M., Rzepecka-Stojko A., Balwierz R., Stojko J. (2020). Bee Products in Dermatology and Skin Care. Molecules.

[6] Farooqui T., Farooqui A.A. (2012). Beneficial effects of propolis on human health and neurological diseases. Front. Biosci. Elite Ed..

[7] Boeing T., Mejía J.A.A., Ccana-Ccapatinta G.V., Mariott M., Melo Vilhena de Andrade Fonseca Da Silva R.C., de Souza P., Mariano L.N.B., Oliveira G.R., da Rocha I.M., da Costa G.A. (2021). The gastroprotective effect of red propolis extract from Northeastern Brazil and the role of its isolated compounds. J. Ethnopharmacol..

[8] Esposito C., Garzarella E.U., Bocchino B., D’Avino M., Caruso G., Buonomo A.R., Sacchi R., Galeotti F., Tenore G.C., Zaccaria V. (2021). A standardized polyphenol mixture extracted from poplar-type propolis for remission of symptoms of uncomplicated upper respiratory tract infection (URTI): A monocentric, randomized, double-blind, placebo-controlled clinical trial. Phytomedicine.

[9] Oryan A., Alemzadeh E., Moshiri A. (2018). Potential role of propolis in wound healing: Biological properties and therapeutic activities. Biomed. Pharmacother..

[10] Anjum S.I., Ullah A., Khan K.A., Attaullah M., Khan H., Ali H., Bashir M.A., Tahir M., Ansari M.J., Ghramh H.A. (2019). Composition and functional properties of propolis (bee glue): A review. Saudi J. Biol. Sci..

[11] Santos L.M., Da Fonseca M.S., Sokolonski A.R., Deegan K.R., Araújo R.P.C., Umsza-Guez M.A., Barbosa J.D.V., Portela R.D., Machado B.A.S. (2020). Propolis: Types, composition, biological activities, and veterinary product patent prospecting. J. Sci. Food Agric..

[12] Shahinozzaman M., Basak B., Emran R., Rozario P., Obanda D.N. (2020). Artepillin C: A comprehensive review of its chemistry, bioavailability, and pharmacological properties. Fitoterapia.

[13] Chiu H.-F., Han Y.-C., Shen Y.-C., Golovinskaia O., Venkatakrishnan K., Wang C.-K. (2020). Chemopreventive and Chemotherapeutic Effect of Propolis and Its Constituents: A Mini-review. J. Cancer Prev..

[14] Przybyłek I., Karpiński T.M. (2019). Antibacterial Properties of Propolis. Molecules.

[15] Erdemli H., Akyol S., Armutcu F., Akyol O. (2015). Antiviral Properties of Caffeic Acid Phenethyl Ester and Its Potential Application. J. Intercult. Ethnopharmacol..

[16] Ota C., Unterkircher C., Fantinato V., Shimizu M.T. (2001). Antifungal activity of propolis on different species of Candida. Mycoses.

[17] Song M.-Y., Lee D.-Y., Kim E.-H. (2020). Anti-inflammatory and anti-oxidative effect of Korean propolis on Helicobacter pylori-induced gastric damage in vitro. J. Microbiol..

[18] Nani B.D., Sardi J.D.C.O., Lazarini J.G., Silva D.R., Massariolli A.P., Cunha T.M., De Alencar S.M., Franchin M., Rosalen P.L. (2020). Anti-inflammatory and anti-Candida Effects of Brazilian Organic Propolis, a Promising Source of Bioactive Molecules and Functional Food. J. Agric. Food Chem..

[19] Wang J., Bhargava P., Yu Y., Sari A.N., Zhang H., Ishii N., Yan K., Zhang Z., Ishida Y., Terao K. (2020). Novel Caffeic Acid Phenethyl Ester-Mortalin Antibody Nanoparticles Offer Enhanced Selective Cytotoxicity to Cancer Cells. Cancers.

[20] Münstedt K., Männle H. (2020). Bee products and their role in cancer prevention and treatment. Complement. Ther. Med..

[21] Maruta H., He H. (2020). PAK1-blockers: Potential Therapeutics against COVID-19. Med. Drug Discov..

[22] Liu X., Du Q., Tian C., Tang M., Jiang Y., Wang Y., Cao Y., Wang Z., Wang Z., Yang J. (2021). Discovery of CAPE derivatives as dual EGFR and CSK inhibitors with anticancer activity in a murine model of hepatocellular carcinoma. Bioorg. Chem..

[23] Wadhwa R., Nigam N., Bhargava P., Dhanjal J.K., Goyal S., Grover A., Sundar D., Ishida Y., Terao K., Kaul S.C. (2016). Molecular Characterization and Enhancement of Anticancer Activity of Caffeic Acid Phenethyl Ester by γ Cyclodextrin. J. Cancer.

[24] Sari A.N., Bhargava P., Dhanjal J.K., Putri J.F., Radhakrishnan N., Shefrin S., Ishida Y., Terao K., Sundar D., Kaul S.C. (2020). Combination of Withaferin-A and CAPE Provides Superior Anticancer Potency: Bioinformatics and Experimental Evidence to Their Molecular Targets and Mechanism of Action. Cancers.

[25] Jin U.-H., Song K.-H., Motomura M., Suzuki I., Gu Y.-H., Kang Y.-J., Moon T.-C., Kim C.-H. (2008). Caffeic acid phenethyl ester induces mitochondria-mediated apoptosis in human myeloid leukemia U937 cells. Mol. Cell. Biochem..

[26] Lee Y.-J., Kuo H.-C., Chu C.-Y., Wang C.-J., Lin W.-C., Tseng T.-H. (2003). Involvement of tumor suppressor protein p53 and p38 MAPK in caffeic acid phenethyl ester-induced apoptosis of C6 glioma cells. Biochem. Pharmacol..

[27] Cho M.S., Park W.S., Jung W.-K., Qian Z.-J., Lee D.-S., Choi J.-S., Lee D.-Y., Park S.-G., Seo S.-K., Kim H.-J. (2014). Caffeic acid phenethyl ester promotes anti-inflammatory effects by inhibiting MAPK and NF-κB signaling in activated HMC-1 human mast cells. Pharm. Biol..

[28] Szliszka E., Mertas A., Czuba Z.P., Krol W. (2013). Inhibition of Inflammatory Response by Artepillin C in Activated RAW264.7 Macrophages. Evid. Based Complement. Altern. Med..

[29] Shao B., Mao L., Shao J., Huang C.-H., Qin L., Huang R., Sheng Z.-G., Cao D., Zhang Z.-Q., Lin L. (2020). Mechanism of synergistic DNA damage induced by caffeic acid phenethyl ester (CAPE) and Cu(II): Competitive binding between CAPE and DNA with Cu(II)/Cu(I). Free Radic. Biol. Med..

[30] Wang X., Bowman P.D., Kerwin S.M., Stavchansky S. (2007). Stability of caffeic acid phenethyl ester and its fluorinated derivative in rat plasma. Biomed. Chromatogr..

[31] Konishi Y., Hitomi Y., Yoshida M., Yoshioka E. (2005). Absorption and Bioavailability of Artepillin C in Rats after Oral Administration. J. Agric. Food Chem..

[32] Wagh V.D. (2013). Propolis: A Wonder Bees Product and Its Pharmacological Potentials. Adv. Pharmacol. Sci..

[33] Bhargava P., Grover A., Nigam N., Kaul A., Doi M., Ishida Y., Kakuta H., Kaul S.C., Terao K., Wadhwa R. (2018). Anticancer activity of the supercritical extract of Brazilian green propolis and its active component, artepillinï¿½C: Bioinformatics and experimental analyses of its mechanisms of action. Int. J. Oncol..

[34] Zabot G.L., Viganó J., Silva E.K. (2021). Low-Frequency Ultrasound Coupled with High-Pressure Technologies: Impact of Hybridized Techniques on the Recovery of Phytochemical Compounds. Molecules.

[35] Baldino L., Scognamiglio M., Reverchon E. (2021). Supercritical CO_2_ Extraction of Organic Solvents from Flunisolide and Fluticasone Propionate. Pharmaceutics.

[36] Baldino L., Adami R., Reverchon E. (2018). Concentration of Ruta graveolens active compounds using SC-CO_2_ extraction coupled with fractional separation. J. Supercrit. Fluids.

[37] Bhargava P., Kumari A., Putri J.F., Ishida Y., Terao K., Kaul S.C., Sundar D., Wadhwa R. (2018). Caffeic acid phenethyl ester (CAPE) possesses pro-hypoxia and anti-stress activities: Bioinformatics and experimental evidences. Cell Stress Chaperones.

[38] Yoon A.-R., Gao R., Kaul Z., Choi I.-K., Ryu J., Noble J.R., Kato Y., Saito S., Hirano T., Ishii T. (2011). MicroRNA-296 is enriched in cancer cells and downregulates p21WAF1 mRNA expression via interaction with its 3′ untranslated region. Nucleic Acids Res..

[39] Putri J.F., Bhargava P., Dhanjal J.K., Yaguchi T., Sundar D., Kaul S.C., Wadhwa R. (2019). Mortaparib, a novel dual inhibitor of mortalin and PARP1, is a potential drug candidate for ovarian and cervical cancers. J. Exp. Clin. Cancer Res..

[40] Majumder D., Nath P., Debnath R., Maiti D. (2021). Understanding the complicated relationship between antioxidants and carcinogenesis. J. Biochem. Mol. Toxicol..

[41] Sosa V., Moliné T., Somoza R., Paciucci R., Kondoh H., Lleonart M.E. (2013). Oxidative stress and cancer: An overview. Ageing Res. Rev..

[42] Konar A., Kalra R.S., Chaudhary A., Nayak A., Guruprasad K.P., Satyamoorthy K., Ishida Y., Terao K., Kaul S.C., Wadhwa R. (2020). Identification of Caffeic Acid Phenethyl Ester (CAPE) as a Potent Neurodifferentiating Natural Compound That Improves Cognitive and Physiological Functions in Animal Models of Neurodegenerative Diseases. Front. Aging Neurosci..

[43] Zhang J., Ding C., Zhang S., Xu Y. (2020). Neuroprotective effects of astaxanthin against oxygen and glucose deprivation damage via the PI3K/Akt/GSK3β/Nrf2 signalling pathway in vitro. J. Cell. Mol. Med..

[44] Mattson M.P., Arumugam T.V. (2018). Hallmarks of Brain Aging: Adaptive and Pathological Modification by Metabolic States. Cell Metab..

[45] Kumar A., Konar A., Garg S., Kaul S.C., Wadhwa R. (2021). Experimental evidence and mechanism of action of some popular neuro-nutraceutical herbs. Neurochem. Int..

[46] Sayed E.A., Badr G., Hassan K.A.-H., Waly H., Ozdemir B., Mahmoud M.H., Alamery S. (2020). Induction of liver fibrosis by CCl4 mediates pathological alterations in the spleen and lymph nodes: The potential therapeutic role of propolis. Saudi J. Biol. Sci..

[47] Ali M.A., Menze E.T., Tadros M.G., Tolba M.F. (2020). Caffeic acid phenethyl ester counteracts doxorubicin-induced chemobrain in Sprague-Dawley rats: Emphasis on the modulation of oxidative stress and neuroinflammation. Neuropharmacology.

[48] Choromanska A., Saczko J., Kulbacka J. (2020). Caffeic Acid Phenethyl Ester Assisted by Reversible Electroporation—In Vitro Study on Human Melanoma Cells. Pharmaceutics.

[49] Spanidi E., Karapetsas A., Voulgaridou G.-P., Letsiou S., Aligiannis N., Tsochantaridis I., Kynigopoulos S., Lambropoulou M., Mourtzinos I., Pappa A. (2021). A New Controlled Release System for Propolis Polyphenols and Its Biochemical Activity for Skin Applications. Plants.

[50] Ishida Y., Gao R., Shah N., Bhargava P., Furune T., Kaul S.C., Terao K., Wadhwa R. (2018). Anticancer Activity in Honeybee Propolis: Functional Insights to the Role of Caffeic Acid Phenethyl Ester and Its Complex With γ-Cyclodextrin. Integr. Cancer Ther..

[51] Shimizu K., Ashida H., Matsuura Y., Kanazawa K. (2004). Antioxidative bioavailability of artepillin C in Brazilian propolis. Arch. Biochem. Biophys..

[52] Ni J., Wu Z., Meng J., Zhu A., Zhong X., Wu S., Nakanishi H. (2017). The Neuroprotective Effects of Brazilian Green Propolis on Neurodegenerative Damage in Human Neuronal SH-SY5Y Cells. Oxidative Med. Cell. Longev..

[53] Park J.W., Sung M.S., Ha J.Y., Guo Y., Piao H., Heo H., Park S.W. (2020). Neuroprotective Effect of Brazilian Green Propolis on Retinal Ganglion Cells in Ischemic Mouse Retina. Curr. Eye Res..

