# Supplementary material for: Molecular Insights into the Antistress Potentials of Brazilian Green Propolis Extract and Its Constituent Artepillin C
Source: Molecules. 2021 Dec 23;27(1):80. doi: 10.3390/molecules27010080 (PMC8746355; doi:10.3390/molecules27010080)
Supplement: Supplementary file 1 [file molecules-27-00080-s001.zip › molecules-1514258-supplementary.pdf]

molecules

MDPI

Supplementary File

Experimental evidence to the anti-stress potentials of super-critical extract of Brazilian green propolis and its constituent Artepillin C

Ashish Kaul[1,2], Raviprasad Kuthethur[1,3], Yoshiyuki Ishida[4], Keiji Terao[4] and Renu Wadhwa[1,2*], Sunil C Kaul[1*]

[1]AIST-INDIA DAILAB, DBT-AIST International Center for Translational & Environmental Research (DAICEN-TER), National Institute of Advanced Industrial Science & Technology (AIST), Tsukuba 305-8565, Japan
[2]School of Integrative & Global Majors, University of Tsukuba 305-8577, Japan
[3]Department of Cell and Molecular Biology, Manipal School of Life Sciences, Manipal Academy of Higher Education (MAHE), Manipal 576-104, India
[4]CycloChem Co., Ltd., 7-4-5 Minatojima-minamimachi, Chuo-ku, Kobe 650-0047, Japan
* Correspondence: renu-wadhwa@aist.go.jp and s-kaul@aist.go.jp

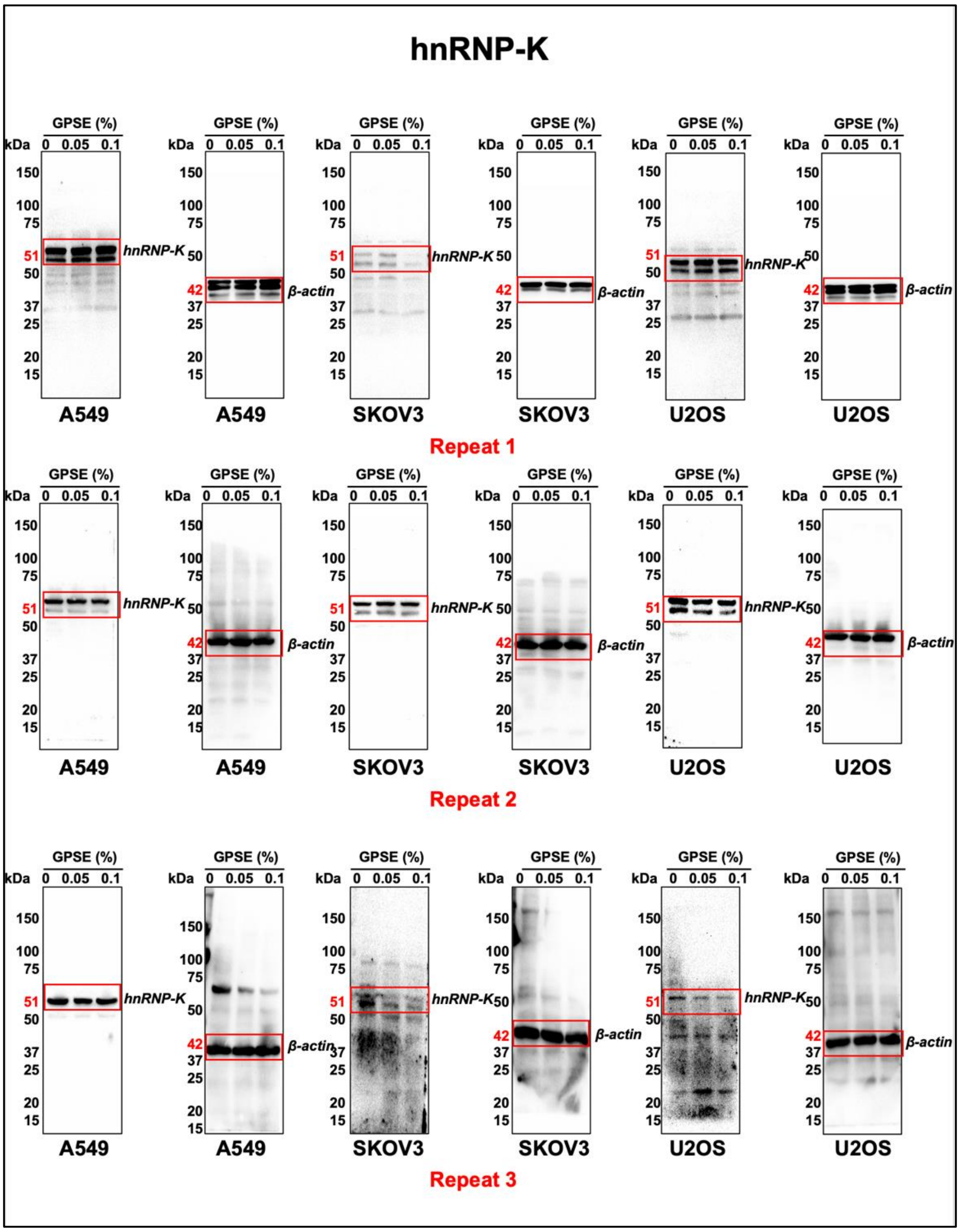

Figure S1. Western blot of protein of interest (hnRNP-K) in three cell lines A549, SKOV3, U2OS and its respective β-actin expression presented in Figure 2B.

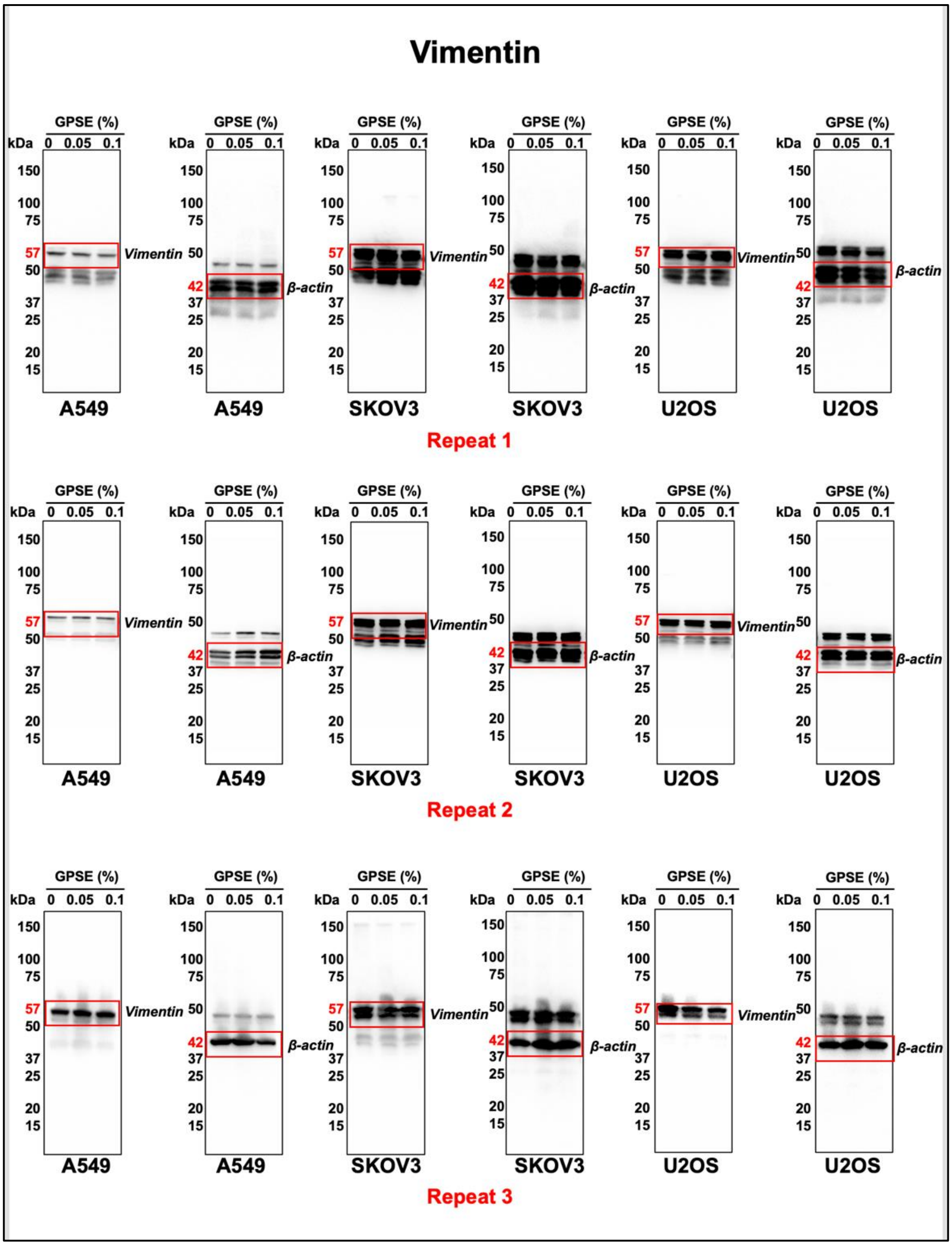

Figure S2. Western blot of protein of interest (Vimentin) in three cell lines A549, SKOV3, U2OS and its respective β-actin expression presented in Figure 2B.

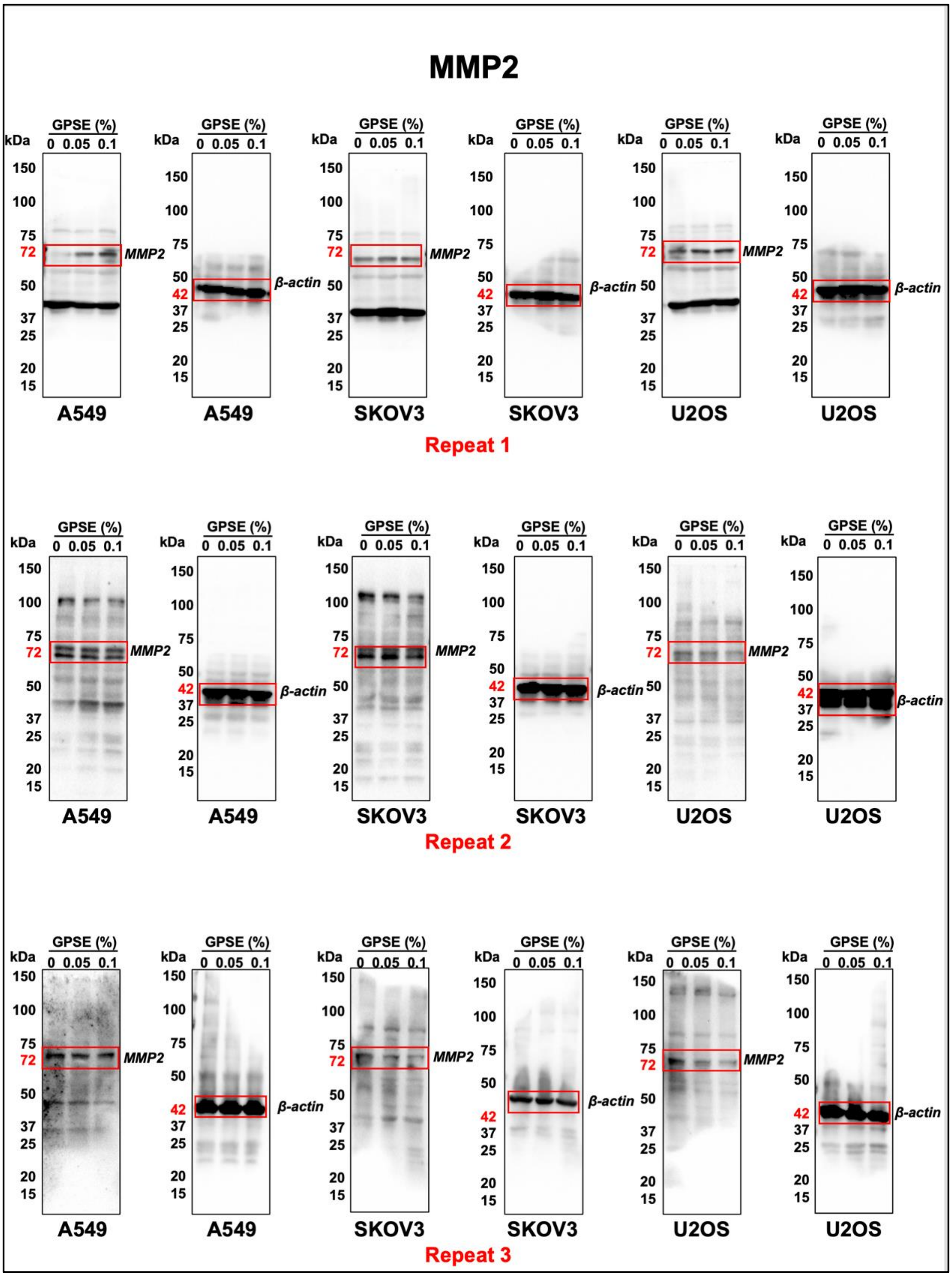

Figure S3. Western blot of protein of interest (MMP2) in three cell lines A549, SKOV3, U2OS and its respective β-actin expression presented in Figure 2B.

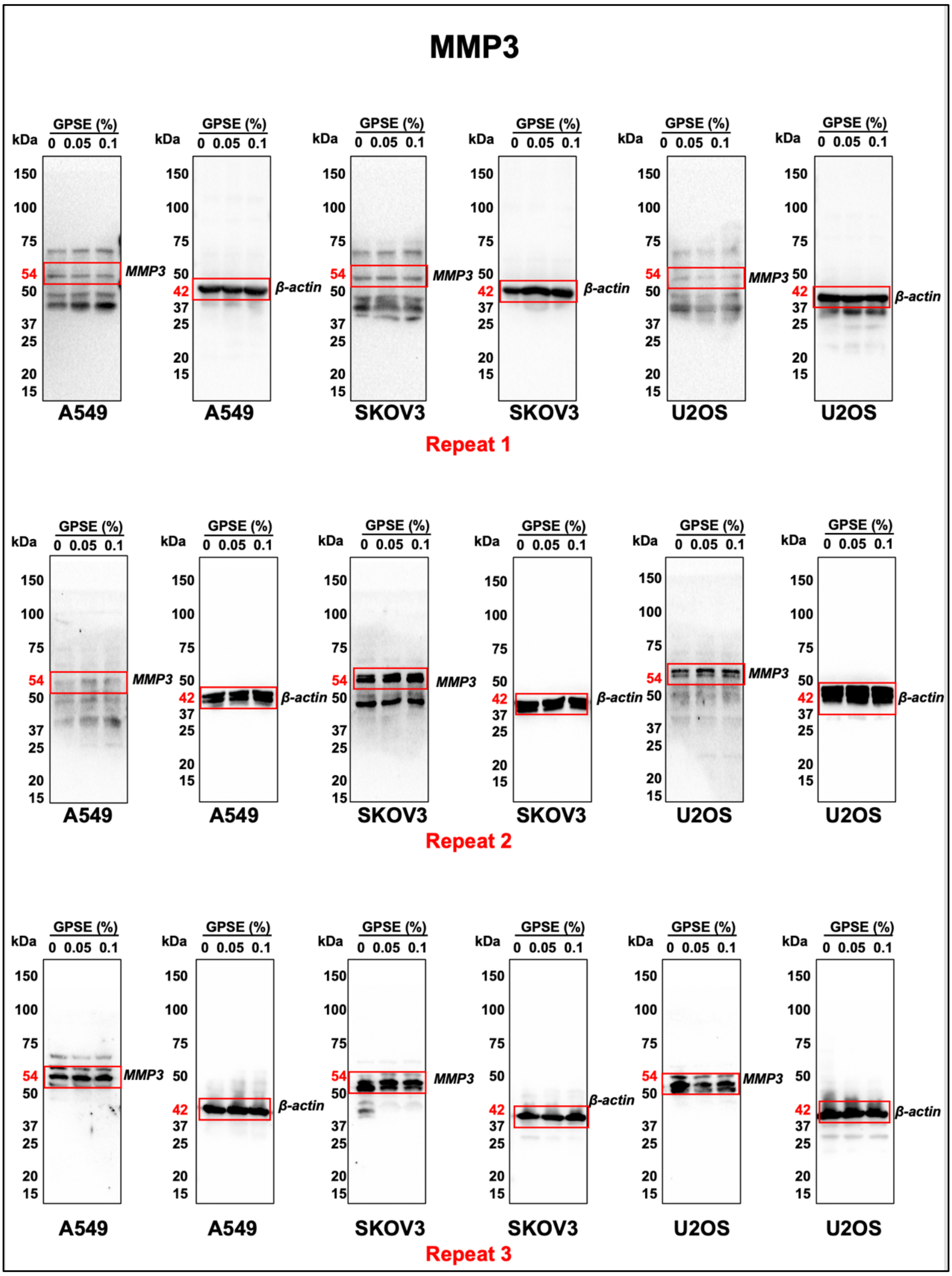

Figure S4. Western blot of protein of interest (MMP3) in three cell lines A549, SKOV3, U2OS and its respective β-actin expression presented in Figure 2B.

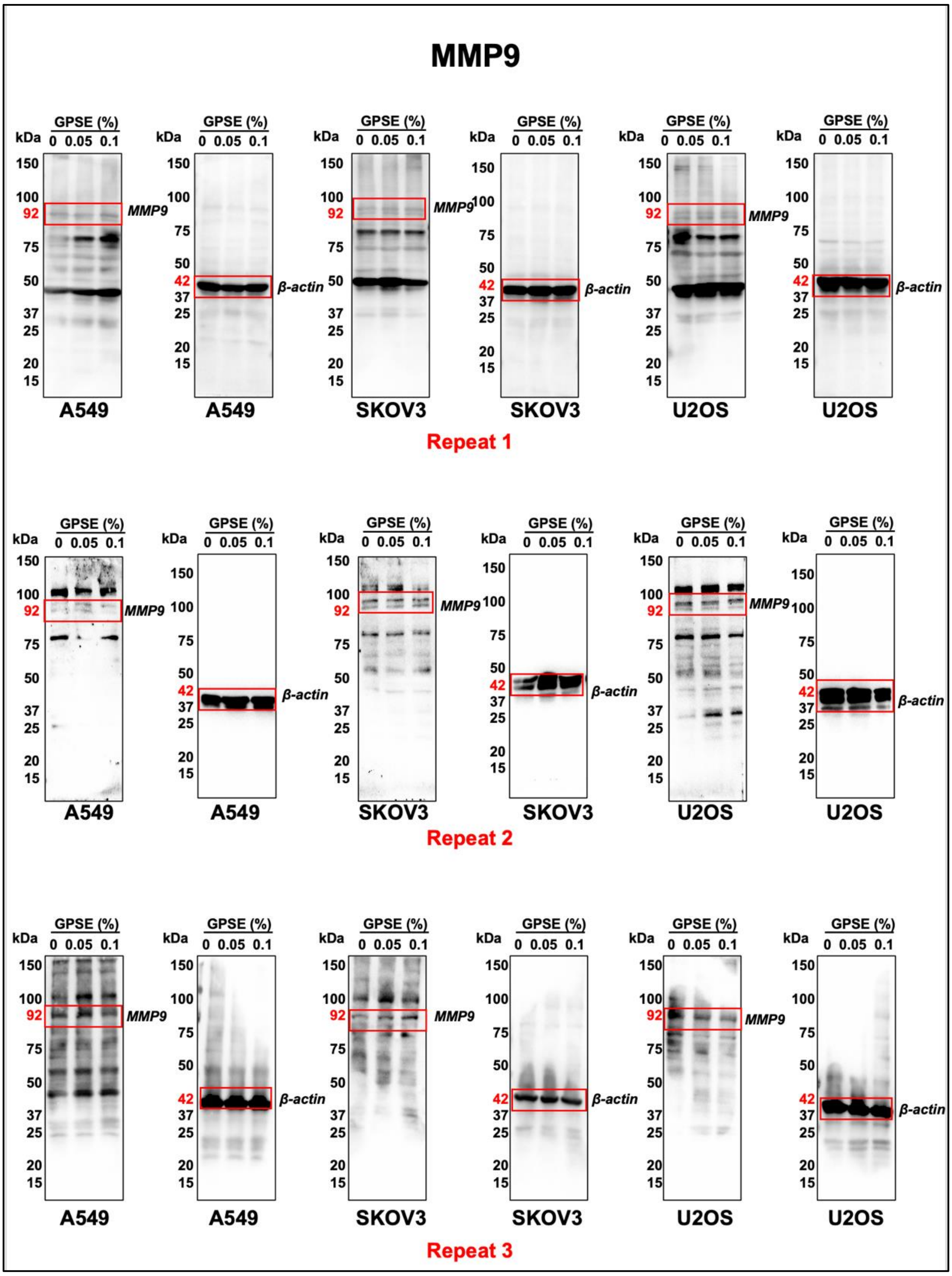

Figure S5. Western blot of protein of interest (MMP9) in three cell lines A549, SKOV3, U2OS and its respective β-actin expression presented in Figure 2B.

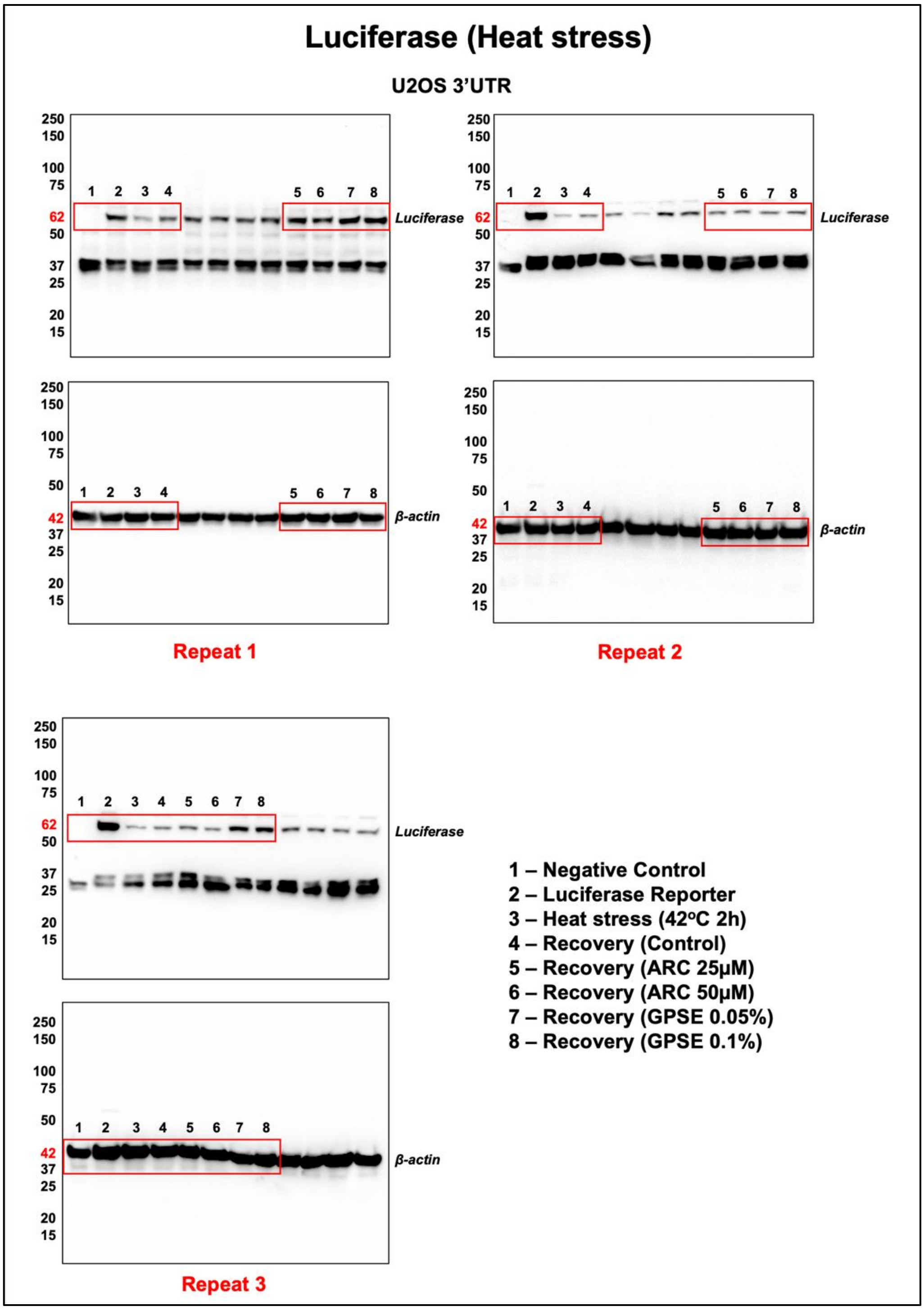

Figure S6. Western blot of protein of interest (Luciferase) and its respective β-actin expression presented in Figure 3C.

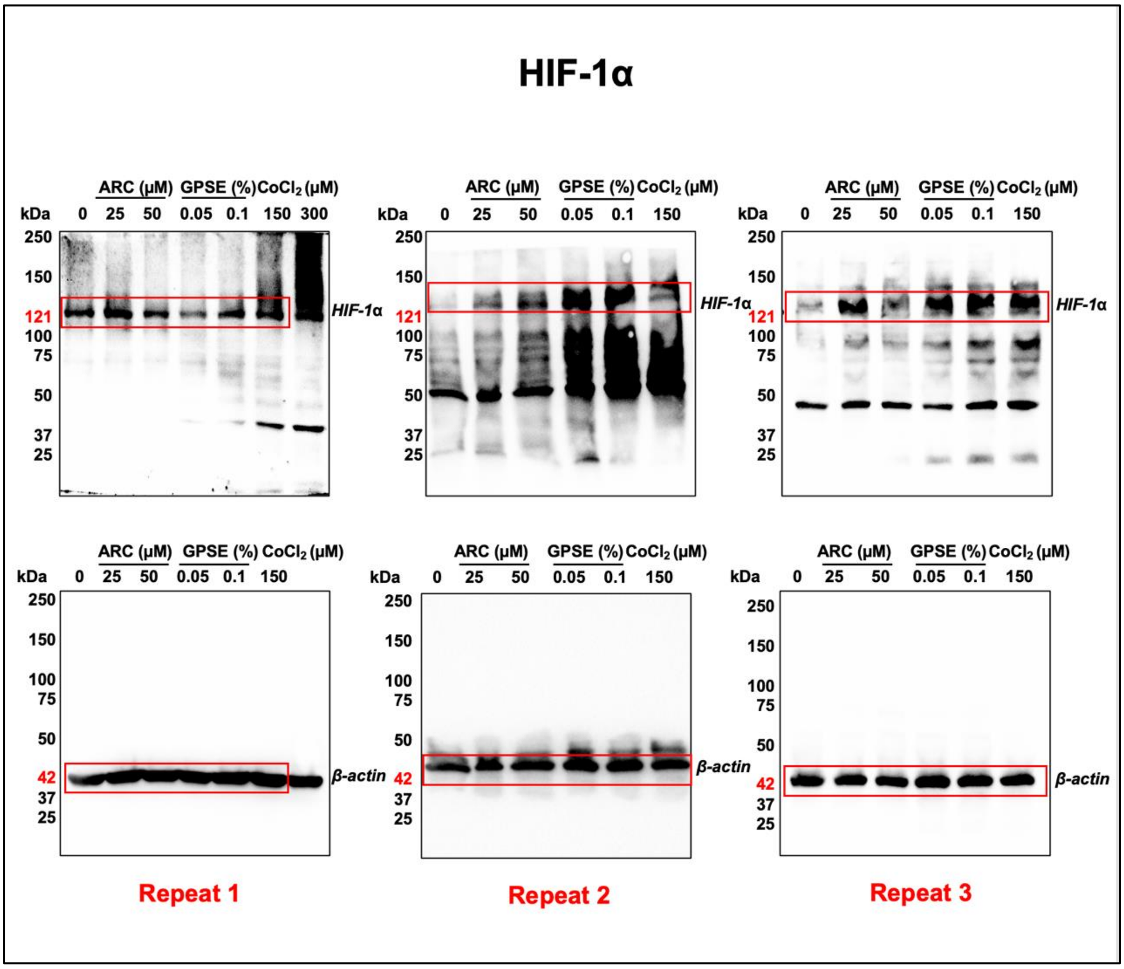

Figure S7. Western blot of protein of interest (HIF-1α) and its respective β-actin expression presented in Figure 4B.

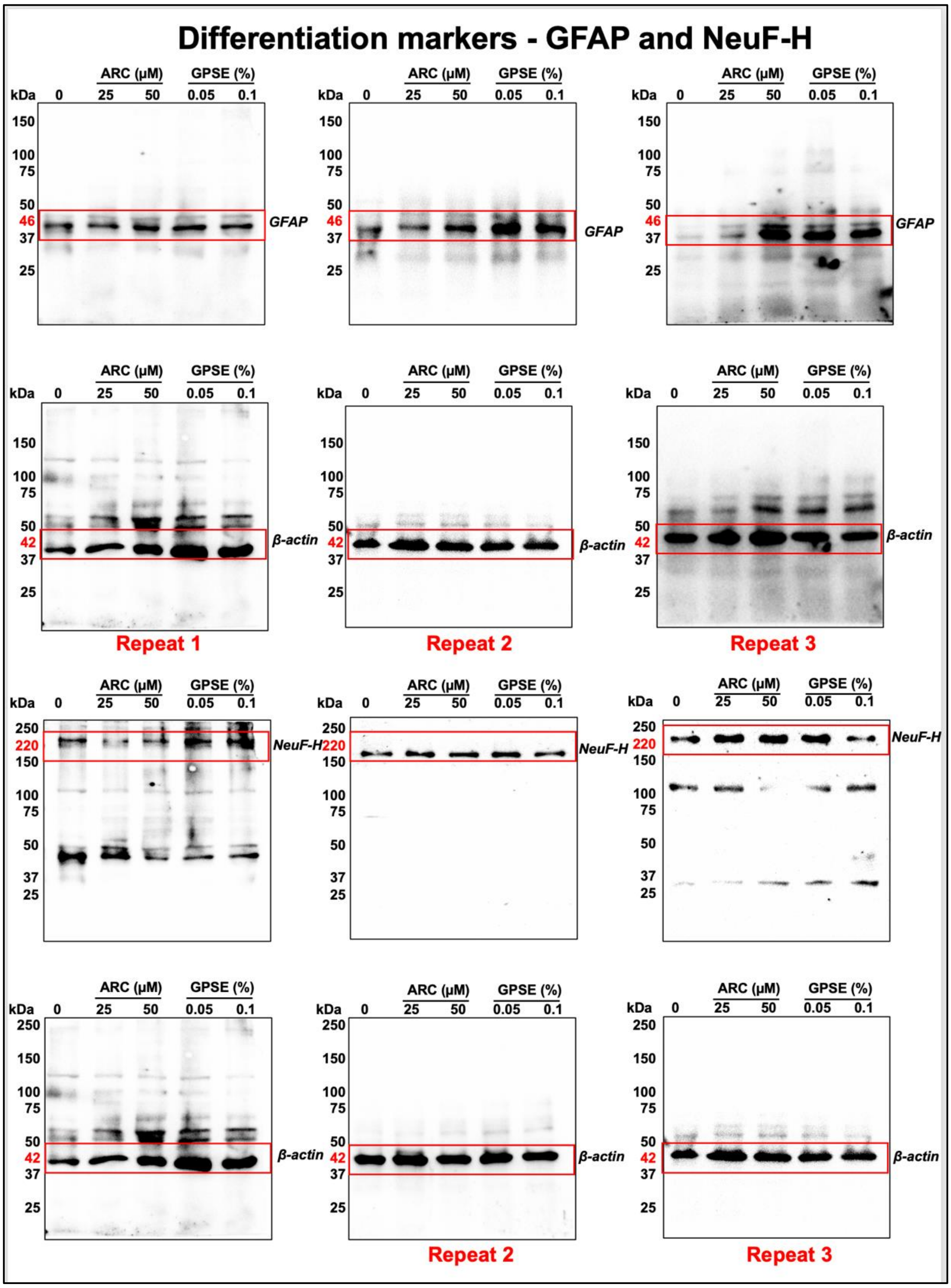

Figure S8. Western blot of proteins of interest (GFAP and NeuF-H) and their respective β-actin expression presented in Figure 5B.

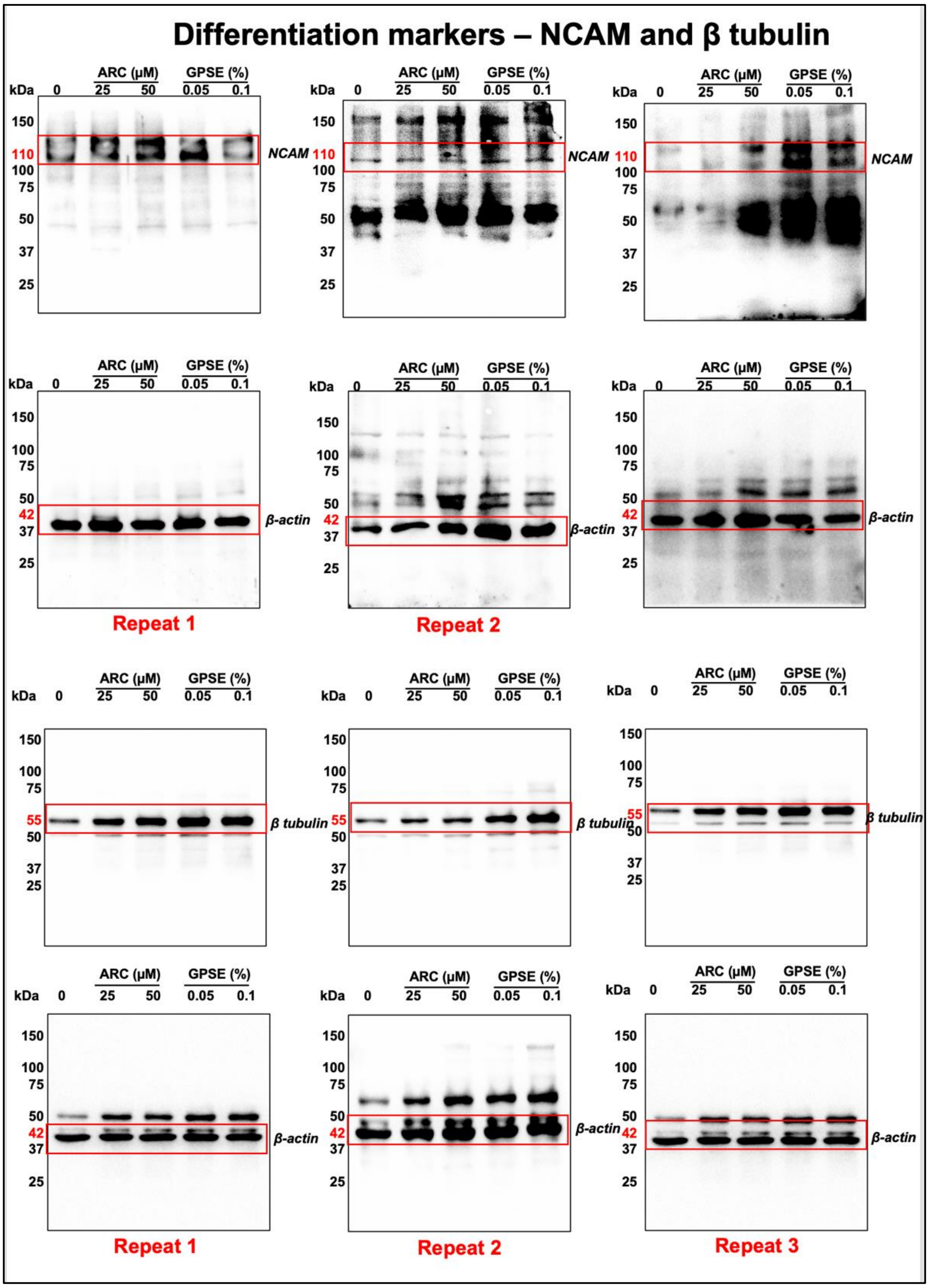

Figure S9. Western blot of proteins of interest (NCAM and β tubulin) and their respective β-actin expression presented in Figure 5B.

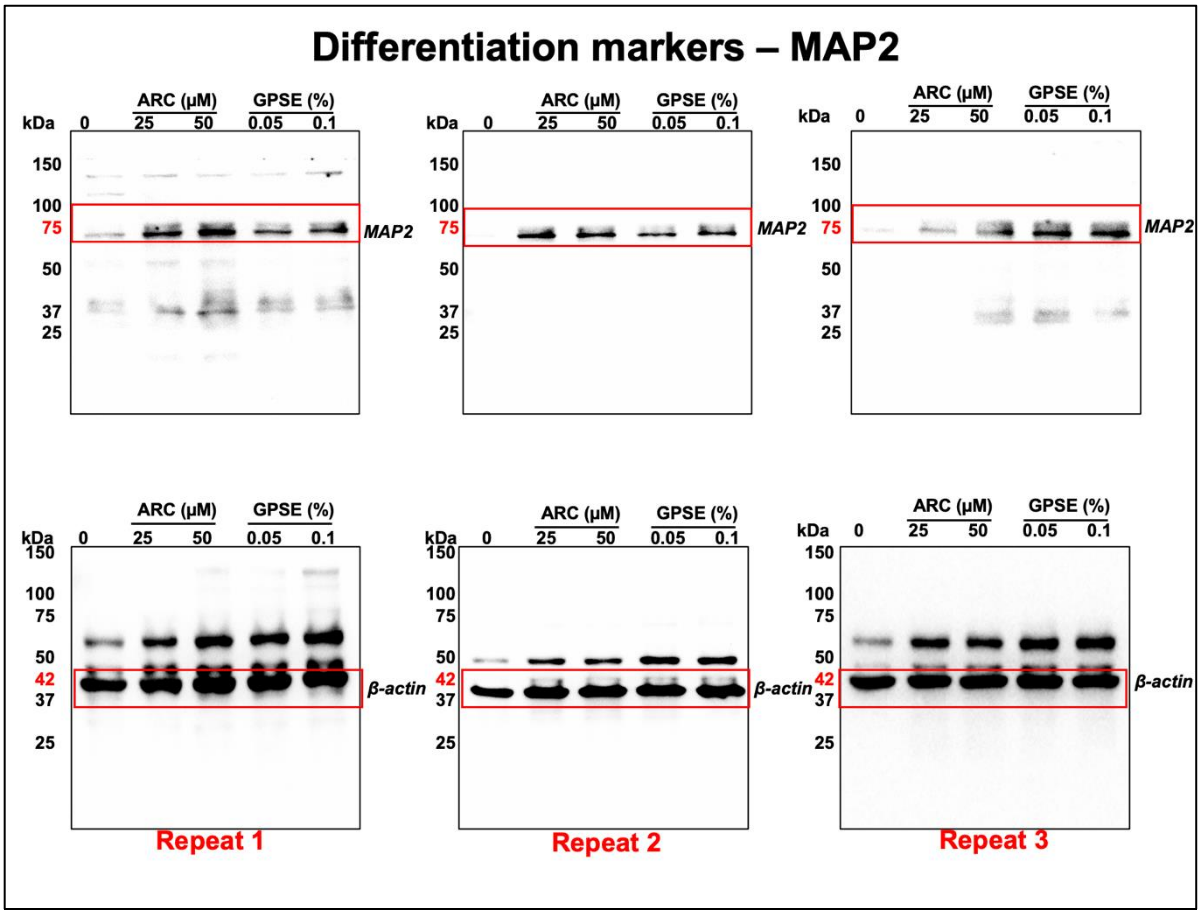

Figure S10. Western blot of protein of interest (MAP2) and its respective β-actin expression presented in Figure 5B.
